# Efficacy of a gB + gD-based subunit vaccine and the adjuvant granulocyte-macrophage colony stimulating factor for pseudorabies virus in rabbits

**DOI:** 10.3389/fmicb.2022.965997

**Published:** 2022-08-03

**Authors:** Zhi Cao, Ke Zhang, Heng Zhang, Hongliang Zhang, Ying Yu, Dehua Yin, Hu Shan, Zhihua Qin

**Affiliations:** ^1^College of Veterinary Medicine, Qingdao Agricultural University, Qingdao, China; ^2^Shandong SINDER Technology Co., Ltd., Qingdao, China

**Keywords:** subunit vaccines, glycoproteins B and D, GM-CSF, protection efficacy, pseudorabies virus

## Abstract

Pseudorabies (PR), which is caused by the pseudorabies virus (PRV), is a severe infectious disease that causes abortions in adult sows and fatal encephalitis in piglets; the disease can occur in pigs of all ages and other mammals, which can lead to significant economic loss around the worldwide. The new PRV variant invalidated the available commercial attenuated and inactivated vaccines. Consequently, subunit vaccines have been suggested as novel strategies for PR control, while they are usually formulated with adjuvants due to their lower immunogenicity. We aimed to select a safe and efficient adjuvant for subunit vaccines for PR. In our study, glycoprotein B (gB) and glycoprotein D (gD) were expressed based on a baculovirus expression system, and granulocyte-macrophage colony-stimulating factor (GM-CSF) was expressed using an *Escherichia coli* (*E. coli*) expression system; subsequently, a gB + gD subunit vaccine adjuvanted by GM-CSF was constructed. A rabbit model infected with a PRV SD-2017 strain was established, the TCID_50_ and LD_50_ were measured, and the typical clinical symptoms were observed. After a lethal challenge of 5 LD_50_ with a PRV SD-2017 strain, the rabbits exhibited typical clinical symptoms, including itching and high temperature, and histopathology revealed severe inflammation in the brain, which is the dominant target organ of PRV. Rabbits immunized with the gB + gD + GM-CSF subunit vaccines produced higher levels of antibodies than those immunized with gB + gD + ISA 201, which was adjuvanted with a frequently used oil adjuvant. The survival rate of rabbits vaccinated with gB + gD + GM-CSF was 100%, which was superior to that of rabbits vaccinated with gB + gD + ISA 201 (80%), inactivated PRV + GM-CSF (60%) and commercial inactivated vaccine (60%) after challenge with PRV SD-2017. These data suggested that the gB + gD + GM-CSF-based subunit vaccine had good protective efficacy against the PRV SD-2017 strain in rabbits and that GM-CSF could be developed as a candidate adjuvant for use in a vaccine regimen to prevent and even eradicate PR.

## Introduction

Pseudorabies (PR), caused by pseudorabies virus (PRV), is a severe infectious disease characterized by abortions in pregnant pigs, fatal nervous system disorders in piglets, and respiratory illness in growing-fattening pigs, leading to significant economic loss to the swine industry worldwide (Freuling et al., 2017; Wang et al., 2018; Jiang et al., 2020). In recent decades, the PRV Bartha-K61 vaccine has been used to protect susceptible herds from PRV infection, and the prevalence of PR was eliminated-completely in the United States and some European countries from their domestic herds (Pannett et al., 1999; Hahn et al., 2010; Wu et al., 2013; Tan et al., 2021). However, a novel PRV variant emerged in pig farms immunized with the Bartha-K61 vaccine and subsequently spread into other countries (including China) in a short time, resulting in substantial economic losses and sharply impeding the development of the swine industry (An et al., 2013; Wu et al., 2013). This event implies that the Bartha-K61 vaccine did not provide complete protection from the virus and that the exploitation of novel veterinary vaccines based on circulating variants is a prerequisite (Wang et al., 2015).

The causative agent, PRV, which belongs to subfamily alpha-Herpesvirinae, is a double-stranded DNA virus containing at least 72 genes encoding 11 different glycoproteins in the viral envelope (gB, gC, gD, gE, gG, gH, gI, gK, gL, gM, and gN) (Li et al., 2017). gB and gD are not primary glycoproteins of PRV for virion attachment to the surface of host cells but rather immunogenic proteins that stimulate the production of neutralizing antibodies against PRV infection (Mettenleiter, 2000; Aschner and Herold, 2021). gB is indispensable for viral entry and spread between cells, while gD is required for receptors binding, stabilizing the interaction of virions and cells, and further activating gB to become fusion competent (Mettenleiter, 1996; He et al., 2019).

Due to their well-defined components with excellent safety, subunit vaccines are considered advantageous for the control of PR over common inactivated and attenuated vaccines, thereby attracting researchers’ focus to protect animal welfare and reduce public health costs (Tan et al., 2021). The differentiation of vaccinated animals (DIVA) from field virus-infected animals could be realized by inoculating with subunit vaccines in conjunction with serological diagnostic tests, which has the potential to eradicate PR disease without the risk of virulence reversion (van Oirschot et al., 1990). Furthermore, there is no latent infection for animals vaccinated by subunit vaccines, which is more appropriate for pregnant animals than attenuated live vaccines.

Accumulating evidence shows that gB and gD are crucial proteins that induce humoral and cellular immune responses and are regarded as attractive targets for the exploration of novel types of vaccines (Mukamoto et al., 1991; Mettenleiter, 1996; Han et al., 2008). It has been reported that antibodies against gB and gD can protect pigs from lethal infection with PRV; however, few available commercial subunit vaccines targeting gB and gD particles have been tested, although abundant trial data exist (Li et al., 2017). Nevertheless, limited by lower immunogenicity, the immune effects induced by subunit vaccines are usually insufficient; therefore, the selection of an adjuvant is essential to improve immune effects.

Granulocyte-Macrophage Colony-Stimulating Factor (GM-CSF), a proinflammatory cytokine, helps elicit robust antiviral and antitumor efficacy in preclinical trials, thereby regulating the development and functions of dendritic cell (DC) subsets, as well as the activation of T lymphocytes (van de Laar et al., 2012). Similar to IL-12, recent *in vivo* experiment-based information about GM-CSF is proving that it is a usable adjuvant for human or animal vaccines (Zhao et al., 2018). Thus, GM-CSF could be regarded as a promising adjuvant for subunit vaccine studies.

To develop more effective subunit vaccines against the PRV SD-2017 strain, gB and gD were expressed based on the baculovirus expression system; furthermore, an efficient adjuvant was selected to enhance the immune response to the subunit vaccines. In our study, rabbits were employed to explore the immunoprotective capacity of the gB + gD subunit vaccines and GM-CSF adjuvant. The clinical symptoms, mortality, neutralizing antibody (NA) titers, gB- and gD- specific antibody levels, and pathology were assessed. The results indicated that gB + gD + GM-CSF subunit vaccines induced a robust immune response and provided efficient protection for rabbits, and that GM-CSF may be a potential candidate adjuvant for subunit vaccines.

## Materials and methods

### Protein expression and purification

The extracellular domain sequence of the gD gene and the N-terminal sequence of the gB gene of the PRV strain (GenBank: MT949536.1) were retrieved from the GenBank database and amplified by PCR utilizing primers designed by Primer 5. The PCR products were cloned into the pFastBac HT-B vector, named pFastBac-gB and pFastBac-gD, which were synthesized by Shanghai Sangn Biotech (China). All recombinant plasmids were transformed into *E. coli* DH10Bac™ (Takara, Japan) competent cells to harvest the recombinant baculovirus shuttle plasmids according to the Bac-To-Bac Baculovirus Expression System (BEVS) directions. The recombinant baculovirus shuttle plasmids were transinfected into Sf9 cells with Cell Infection II Reagent (Gibco) to obtain soluble gB and gD proteins. Soluble gB and gD proteins were purified using a Ni column, and the concentrations of gB and gD were measured using a BCA Protein Assay Kit. Proteins were identified by SDS-PAGE and Western blotting.

### Virus and animals

The pseudorabies virus (PRV) SD-2017 strain was isolated by Shandong Key Lab of Preventive Veterinary Medicine, Qingdao Agricultural University (QAU), China, and was identified as a PRV mutant (data unpublished). PRV was propagated in porcine kidney cells (PK-15 cells) and stored at –80°C. The PK-15 cells were propagated in Dulbecco’s modified Eagle’s medium (DMEM; HyClone) supplemented with 10% fetal bovine serum (FBS; Gibco).

Three-month-old rabbits were purchased from Qingdao Kangda Biological Co., Ltd. (Shandong, China). All rabbit experiments were performed at Shandong SINDER Technology Co., Ltd. (SINDER, Shandong), and in accordance with the protocols approved by the Animal Care and Ethics Committee of the SINDER, under the number 20180024.

### Adjuvants

ISA 201 oil adjuvant (SEPPIC, France) and GM-CSF were used as candidate adjuvants of subunit vaccine, of which GM-CSF was expressed by an *E. coli* expression system. Briefly, sequences of porcine-origin GM-CSF genes (GenBank: U67175.1) were retrieved from the GenBank database, and whole gene synthesis was performed by Shanghai Sangon Biotech, with the gene cloned into the pET-32a vector (Takara, China). The recombinant plasmid was transformed into *E. coli* BL21 (DE3) competent cells (Takara), and IPTG (Takara) was used to induce the generation of soluble proteins. GM-CSF was purified using a His-tagged protein purification kit (CWBio, China) and the concentrations of gB and gD were measured using a BCA Protein Assay Kit. The expressed product was identified by SDS-PAGE.

### Determination of TCID_50_

PK-15 cells were cultured in DMEM containing 10% FBS, and 100 μL (2 × 10^5^) of cells were seeded in 96-well plates and incubated at 37°C in 5% CO_2_. Then, 100 μL/well of tenfold-diluted (from 10^–1^ to 10^–10^) PRV in DMEM was added to single-layer PK-15 cells in 96-well plates for eight replicates of each dilution, and 100 μL/well DMEM was added as a control group and incubated at 37°C in 5% CO_2_. After incubation for 2 h, the supernatant was discarded, and the 96-well plate was washed twice with PBS. Subsequently, DMEM supplemented with 2% FBS was added to the 96-well plate, and the plate was placed at 37°C in 5% CO_2_ to produce cytopathic effects (CPEs). CPEs were observed and recorded for approximately 5 days, and TCID_50_ was calculated based on the Reed-Muench method (Table 1).

**TABLE 1:** CPEs of PRV-infected PK-15 cells during the 5-day observation period.

Dilution	CPE	No CPE	Total		Percentage of CPE (%)
			CPE	No CPE	
10^–1^	8	0	62	0	100
10^–2^	8	0	54	0	100
10^–3^	8	0	46	0	100
10^–4^	8	0	38	0	100
10^–5^	8	0	30	0	100
10^–6^	7	1	22	1	96.65
10^–7^	7	1	15	2	88.23
10^–8^	5	3	5	5	61.54
10^–9^	2	6	3	11	21.43
10^–10^	1	7	1	18	5.26

### Determination of LD_50_

The PRV SD-2017 strain was diluted with DMEM to 1000 TCID_50_, 500 TCID_50_, 250 TCID_50_, and 125 TCID_50_, and the diluted PRV was stored at –80°C. Twenty-three-month-old unvaccinated rabbits were randomly divided into 4 groups (*n* = 5) and inoculated intranasally with different doses of PRV (1000TCID_50_, 500 TCID_50_, 250 TCID_50_, and 125 TCID_50_). The rectal temperature and survival rate were recorded for 10 days. The LD_50_ was determined based on the Reed-Muench method (Table 2).

**TABLE 2:** Death of rabbits during the 10-day observation period.

Groups	Days post-challenge (Death/Total)									
	1	2	3	4	5	6	7	8	9	10
1000 TCID_50_	0/5	0/5	2/5	4/5	5/5	5/5	5/5	5/5	5/5	5/5
500 TCID_50_	0/5	0/5	0/5	1/5	4/5	4/5	4/5	4/5	4/5	4/5
250 TCID_50_	0/5	0/5	0/5	0/5	0/5	1/5	1/5	1/5	1/5	1/5
125 TCID_50_	0/5	0/5	0/5	0/5	0/5	0/5	0/5	0/5	0/5	0/5

### Vaccine preparation

The compound of gB and gD proteins (each 100 μg) were blended with ISA 201 adjuvant as a water-in-oil vaccine (gB + gD + ISA 201) and with GM-CSF as a water-in-water vaccine (gB + gD + GM-CSF) at a ratio of 1:1 (m/m).

The PRV antigen was inactivated by beta-propiolactone at a final concentration of 0.1% for 24 h at 4°C and then placed in a water bath for 2 h at 37°C for complete inactivation. The inactivated PRV (10^7.29^ TCID_50_) was mixed in GM-CSF adjuvant at a ratio of 1:1 (v/v) as a water-in-water inactivated PRV vaccine (inactivated PRV + GM-CSF).

### Rabbit immunization and sample collection

Three-month-old rabbits (*n* = 25) were randomly divided into five groups. After acclimation, three groups were vaccinated by different types of vaccines as described above. One group was immunized with commercial inactivated vaccine (CIV; Huahong, China) and last group received no immunization as the mock group. After the first immunization for 14 days, a second boost immunization was administered. Blood near the ears was collected for serum separation to assess the levels of gB and gD specific antibodies and neutralizing antibodies at 14 and 28 days post-immunization (dpi).

### Challenge with pseudorabies virus

To observe the positive effect of GM-CSF on the survival rate of rabbits, all vaccinated rabbits were challenged intranasally with 5 LD_50_ of PRV SD-2017 strain after two immunizations. Two rabbits from mock group were challenged as the PRV-challenged group, and another three rabbits from mock group received no injection as the normal group. The rectal temperatures, clinical symptoms, and survival rates were recorded for 14 days. At 14 days post-challenge (dpc), all surviving rabbits were euthanized, and brain, liver, and lung samples were collected for histopathological analysis.

### Detection of antibodies in serum samples

#### gB-specific antibody test

The serum samples described above were separated and tested for the production of gB-specific antibodies with commercial enzyme-linked immunosorbent assay (ELISA) kits (Wuhan Keqian Biology) according to the manufacturer’s instructions.

#### gD-specific antibody test

Recombinant gD protein (200 ng) was diluted in PBS and coated into 96-well ELISA plates overnight at 4°C. Then, 1% BSA was used to block the binding sites for 2 h at 37°C. Both the serum sample and controls were added to the well to combine with coated gD protein for 1 h at 37°C, and an HRP-labeled monoclonal antibody was added for 30 min at room temperature. Finally, the OD_630 *nm*_ value of fluorescence was measured by a microplate reader within 10 min. When the difference between the average value of the negative control, named “N,” and the positive control was equal to or greater than 0.4, the trial was set up. If the quotient between the value of the serum sample, named “S,” and the value of the negative control was less than or equal to 0.6 (S/N ≤ 0.6), the serum sample was judged as “positive”; if the value of S/N was lower, the antibody titers of the serum sample were higher.

#### Neutralizing antibody test

Neutralizing antibodies against the PRV SD-2017 strain were measured by cell tests. Serum samples were heat inactivated for 30 min at 56°C and twofold-diluted (from 2^–1^ to 2^–8^) with DMEM. Then, 500 μL of each dilution of inactivated serum was mixed with an equal volume of PRV SD-2017 strain at a concentration of 100 TCID_50_. 100 μL of the mixture was added to a 96-well plate filled with a single-layer of PK-15 cells, and each dilution was repeated eight times. The virus-positive control well received an equal volume of PRV without serum, and the cell negative control well received an equal volume of DMEM containing 2% FBS. The plate was incubated at 37°C in a 5% CO_2_ atmosphere, and CPEs were observed for approximately 5 days. The neutralizing antibody titer was determined based on the Reed-Muench method.

### Quantification of cytokines in serum samples

IFN-γ and IL-4 levels in serum at 28 dpi were quantified using commercially available ELISA kits according to the manufacturer’s instructions (Nanjing Jiancheng Bioengineering Institute, China).

### Qualification of the pseudorabies virus loads

In total, 100 mg of brain, liver, and lung tissues were cut into tiny pieces and fully ground in sterilized lysis buffer under aseptic conditions. Following complete grinding, the suspension of tissues in PBS was collected and filtered through a 0.22 μm filter after centrifugation at 8,000 rpm at 4°C for 10 min. Genomic DNA from these samples was extracted using a BioFlux Plasmid DNA Mini Extraction Kit (BioFlux, China) and stored at –20°C. Real-time fluorescent quantitative PCR (qPCR) was performed with a probe qPCR mix (with UNG) (Takara) on a qPCR system (Quantitative 5, Applied Biosystems).

### Histopathology

The representative rabbits from each group were dissected, and the brains, lungs, and livers were collected randomly and fixed in 10% neutral formalin at room temperature for 24 h. The fixed tissues were embedded in paraffin wax, stained with hematoxylin and eosin (H&E), and examined by light microscopy.

### Statistical analysis

Data are presented as the mean ± standard deviation (*SD*) of three replicates. Data were analyzed by one-way ANOVA and two-way ANOVA using GraphPad Prism software. Significance is presented as *p < 0.05 and **p < 0.01.

## Results

### Protein expression

**FIGURE 1:**
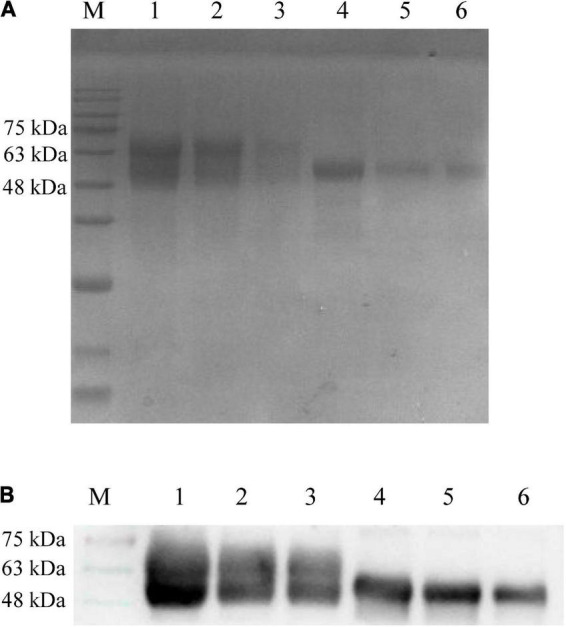
Detection of recombinant proteins gB and gD by SDS-PAGE **(A)** and Western blot **(B)**. **(A)** The culture supernatant of sf9 cells was harvested and purified using a Ni column. The purified recombinant proteins gB and gD were diluted with PBS and mixed with 4 × loading buffer to conduct SDS-PAGE. M: Solarbio 180 Marker. 1: gB (1,500 μg/mL), 2: gB (750 μg/mL), 3: gB (375 μg/mL), 4: gD (300 μg/mL), 5: gD (150 μg/mL), 6: gD (75 μg/mL). **(B)** PRV-positive serum (collected by our laboratory, 1:500) was used as the primary antibody, and HRP-labeled goat anti-rabbit antibody (1:1,000) was used as the secondary antibody. M: Solarbio 180 Marker, 1: gB (1,500 μg/mL), 2: gB (750 μg/mL), 3: gB (375 μg/mL), 4: gD (300 μg/mL), 5: gD (150 μg/mL), and 6: gD (75 μg/mL).

**FIGURE 2:**
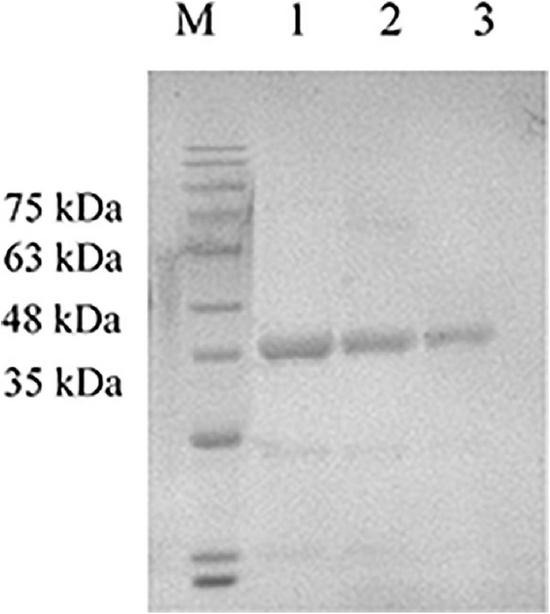
Detection of the expression product GM-CSF by SDS-PAGE. The purified expression product GM-CSF was diluted with PBS and mixed with 4 × loading buffer to conduct SDS-PAGE. M: Solarbio 180 Marker, 1: GM-CSF (2,000 μg/mL), 2: GM-CSF (1,000 μg/mL), and 3: GM-CSF (500 μg/mL).

The gB and gD expression values were calculated at masses for 63 and 55 kDa, respectively (Figure 1), and the concentrations of gB and gD were 3 mg/mL and 300 μg/mL, respectively. GM-CSF expression values were calculated at a mass for 35 kDa (Figure 2), and the concentration of GM-CSF was 2 mg/mL.

### Pseudorabies virus SD-2017 strain-infected rabbit model construction

**FIGURE 3:**
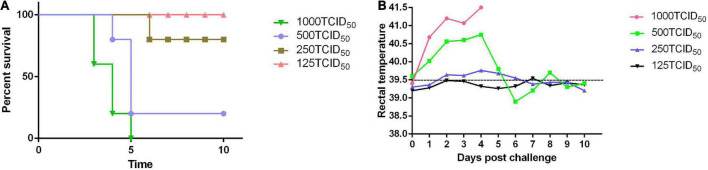
The LD_50_ of the PRV SD-2017 strain was measured in rabbits. Four groups of rabbits were inoculated subcutaneously with different doses of PRV (1000 TCID_50_, 500 TCID_50_, 250 TCID_50_, and 125 TCID_50_). Mortality **(A)** and rectal temperature **(B)** were recorded during the 10-day observation period.

The rabbits were challenged with different doses of the PRV SD-2017 strain, including 1000 TCID_50_, 500 TCID_50_, 250 TCID_50_, and 125 TCID_50_. With the inoculation dose of 1000 TCID_50_, rabbits started to display clinical symptoms at 3 dpc, and all died within 3–5 dpc. The rabbits treated with a 500 TCID_50_ dose exhibited milder PR-associated symptoms at 4 dpc, and the mortality rate was ultimately 80%, while that of the rabbits treated with a 250 TCID_50_ dose was 20%, However, there were no deaths in the 125 TCID_50_ group, and no symptoms appeared (Figure 3A). According to the Reed-Muench method, the LD_50_ of rabbits challenged with the PRV SD-2017 strain was 3.55 × 10^2^ TCID_50_.

Rabbits exhibited slight symptoms at 3 dpc and had a fever of 41°C (Figure 3B). Then, some universal symptoms were observed, including loss of appetite, roughening of the fur, and itching at the injection sites, leading to areas of hair removal. Subsequently, neurological symptoms were gradually induced at 3–5 dpc, such as ataxia and trembling, and rabbits ultimately died at 3–6 dpc.

### Efficacy of a gB + gD-based subunit vaccine and the different adjuvants for pseudorabies virus SD-2017 strain in rabbits

#### Clinical signs post-challenge

**FIGURE 4:**
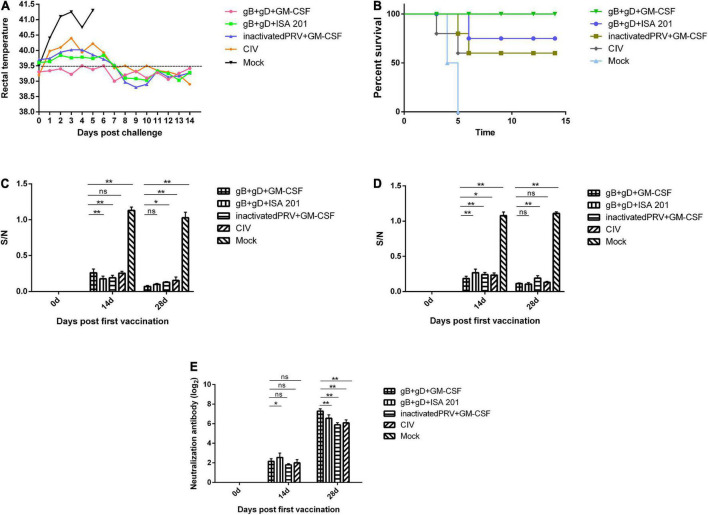
Protection against challenge with the PRV SD-2017 strain in rabbits. Rabbits of three groups were inoculated with gB + gD + GM-CSF (each 100 μg with 200 μg GM-CSF), gB + gD + ISA 201 (each 100 μg with 200 μg of ISA 201), or inactivated PRV + GM-CSF (1 mL of inactivated PRV with 1 mL of ISA 201). One group was vaccinated by commercial inactivated vaccine (CIV) and another group was not injected and served as a mock group. All rabbits were immunized at 0 and 14 days and challenged at 28th day with 5 LD_50_ of the PRV SD-2017 strain. Blood was collected on the 14th and 28th days. Rectal temperature **(A)** was measured during the 14-day observation period, and mortality **(B)** was recorded. gB-specific antibody **(C)**, gD-specific antibody **(D)** and NA titers **(E)** in serum were measured at 14th and 28th days. Data were analyzed by two-way ANOVA using GraphPad Prism software. Significance is presented as *p < 0.05, **p < 0.01. Error bars represent *SD*.

Compared to the normal temperature of 39°C, and the PRV-challenged group maintained a high fever as high as 41°C until death (Figure 4A). The PRV-challenged group displayed typical pathogenesis progression of PR syndrome, including fever, itching, convulsions, and symptoms such as ataxia, and finally died within 5 dpc. However, the immunized groups exhibited milder clinical symptoms, and rabbits immunized with gB + gD + GM-CSF recovered to healthy without inflammation or hemorrhagic points at the injection sites during the 14-day observation period.

#### Mortality examination

Death began to occur on the 4th dpc in the PRV-challenged group, and all two rabbits died within 5 dpc. There were remarkable PR-associated representative clinical symptoms, such as itching and opisthotonos, in all rabbits that succumbed. During the 14-day observation period, there were no deaths in the gB + gD + GM-CSF group (100% survival), while two, one, and two dead rabbits were found in the gB + gD + ISA 201 group (80% survival), inactivated PRV + GM-CSF group (60% survival), and CIV group (60% survival), respectively (Figure 4B).

#### Humoral immune responses post-vaccination

The collected sera in all rabbits immunized with vaccines were positive by ELISA for gB-specific antibodies, as S/N value was lower than 0.6. The S/N values after the second immunization were generally lower than those after the first immunization, which was similar to the results for gD-specific antibodies, reflecting that the serum antibody titers continued to increase steadily. In contrast, the mock group was negative at 14 days post-immunization, as S/N value was higher than 0.7 (Figures 4C,D).

For the gB + gD + GM-CSF and gB + gD + ISA 201 groups, as expected, the antibody levels of both groups of rabbits were higher than those of the inactivated PRV + GM-CSF group and CIV group, which indicated the better immunogenicity of the recombinant proteins. The gB + gD-GM-CSF group exhibited higher antibody titers than the other vaccinated groups (Figures 4C,D).

#### Viral neutralizing responses post-vaccination

We further assessed the neutralizing activity of sera from immunized rabbits using an *in vitro* assay with PK-15 cells. PRV-specific neutralizing antibodies were detected in all immunized rabbit sera, but neutralizing antibodies in the mock group remained negative during the whole study period. On the 14th day after initial immunization, neutralizing antibodies were detected in all vaccinated groups. Subsequently, 14 days after the second boost immunization, the titers of neutralizing antibodies displayed a sharp increase in all vaccinated groups. NAs titers of the gB + gD + GM-CSF group (2^7.28^) were remarkably higher than the gB + gD + ISA 201 group (2^6.56^), inactivated PRV + GM-CSF group (2^5.89^), and CIV group (2^6.1^) (Figure 4E).

#### Cellular immune responses post-vaccination

**FIGURE 5:**
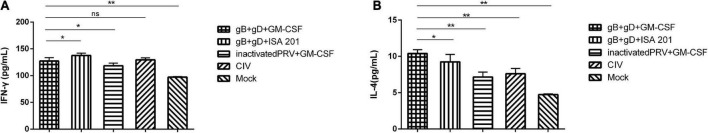
Cellular immune responses analysis in immunized rabbits. IFN-γ **(A)** and IL-4 **(B)** levels in serum at 28 dpi were detected. Data were analyzed by one-way ANOVA using GraphPad Prism software. Significance is presented as *p < 0.05, **p < 0.01. Error bars represent *SD*.

To further investigate the cellular responses of the vaccinated group, ELISA kits were used to quantify the IL-4 and IFN-γ cytokines, which are involved in Th2-biased and Th1-biased cellular responses, respectively. The results showed that IL-4 and IFN-γ levels were increased in the vaccinated groups compared with those in mock group at 28 dpi. Moreover, IL-4 in gB + gD + GM-CSF group was higher than those in other vaccinated groups. IL-4 in gB + gD + GM-CSF group was higher than those in gB + gD + ISA 201 group, which suggested efficacy of GM-CSF for Th2-biased cellular responses (Figure 5).

#### Virus loads in tissues

**FIGURE 6:**
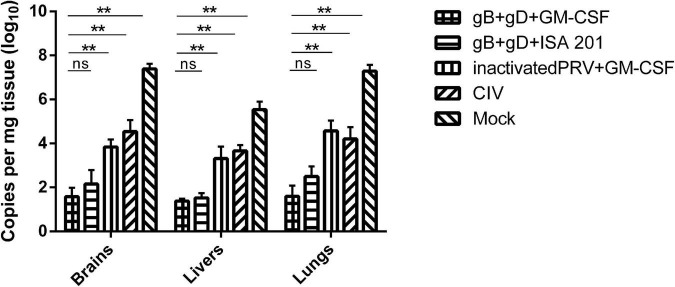
Quantitation of PRV genome in rabbits tissues. PRV DNA was quantified in rabbit tissues using qPCR. PRV loads were shown as copies per mg of tissue. Data are representative of three independent experiments (error bars represent *SD*). Significance is presented as **p < 0.01.

To analyze the PRV levels in different tissues, nucleic acids from the brain, liver, and lung tissues were collected for qPCR analysis to determine the PRV DNA load. The results showed that the DNA load of tissues from rabbits immunized with gB + gD + GM-CSF and gB + gD + ISA 201 were significantly lower than in the PRV-challenged group, whereas viral DNA in tissues from the PRV-challenged group was detected in the brains (10^7.39^ copies/mg), livers (10^5.54^ copies/mg), and lungs (10^7.29^ copies/mg) (Figure 6).

#### Pathological examination

**FIGURE 7:**
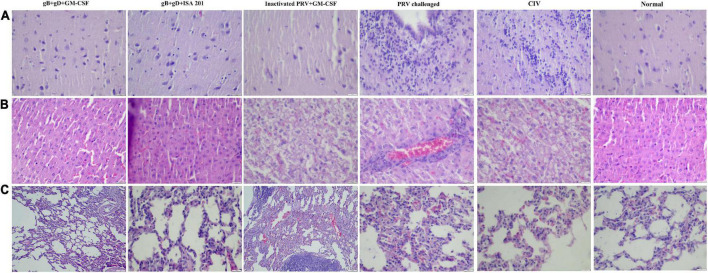
Histopathology examination. In immunized rabbits at 14 days post-challenge, hematoxylin and eosin (H&E) staining was used to detect the pathological lesions of the brains **(A)**, livers **(B)**, and lungs **(C)** from the gB + gD + GM-CSF, gB + gD + ISA 201, inactivated PRV + GM-CSF, PRV-challenged, CIV and the normal groups. Tissues were sectioned into 5-μm slices. Scale bar: 100 μm.

The rabbits immunized with gB + gD + GM-CSF subunit vaccines did not exhibit any pathological lesions, including bleeding points or necrosis (Figures 7A–C, gB + gD + GM-CSF). In contrast, the rabbits challenged with no immunization all had severe hemorrhage and necrosis (Figures 7A–C, PRV challenged). In the brain, which is the vital target organ, there was obvious meningorrhagia (Figure 7A, PRV challenged). Hemorrhagic points occurred in the lung and liver (Figures 7B,C, PRV challenged). Additionally, there was partial lung and liver swelling, and the organ texture became harder compared to that in the vaccinated group. Several rabbits exhibited flatulence. Compared to the gB + gD + GM-CSF group, the gB + gD + ISA 201 group, inactivated PRV + GM-CSF group and CIV group had more serious pathological damage, but the protection from viral damage in the gB + gD + ISA 201 group, inactivated PRV + GM-CSF group and CIV group was better than that in the mock group (Figure 7).

## Discussion

PR is an acute and high-mortality infectious disease characterized by fever, respiratory disorders, and neurological symptoms caused by PRV, which can infect a wide range of mammals and cause nearly 100% mortality (Freuling et al., 2017; Chen et al., 2019; Zhang et al., 2021). In recent years, the outbreak of PR has re-emerged in Bartha-K61-immunized pig herds due to the occurrence of the PRV variants, and the failure of traditional vaccines has led to serious public concerns (Wu et al., 2013; Gu et al., 2015). Studies have shown that gene recombination between vaccinated and field strains results in the emergence of PRV strains, remarkably increasing virulence (Wu et al., 2013; Yu et al., 2014; Ye et al., 2016). Consequently, it is essential to develop novel types of vaccines, and subunit vaccines are a wise choice due to their stability, safety, and well-defined components.

Except for swine and goats, which are susceptible to PRV, rabbits are more susceptible to PRV, and they manifest typical clinical symptoms (Li et al., 2021). Mice usually serve as the desired animal model to conduct basic studies of PRV, while PRV-infected rabbit models are rarely reported. In our previous study, taking the susceptibility of rabbits and the infrequency of the rabbit model into consideration, rabbits were selected as target animals for the immunization trial in our study. The LD_50_ of the PRV SD-2017 strain in rabbits was 3.55 × 10^2^ TCID_50_, indicating the strong virulence of the PRV SD-2017 strain and the high sensitivity of rabbits to the PRV SD-2017 strain. During the initial period, the rectal temperature of all challenged rabbits increased until 4 dpc (Figure 3A). The rabbits of the 1000 TCID_50_ group became restless, itched and scratched at the injection sites from 3 dpc, and died in a short period of time. After 10 days of observation, the rabbits that exhibited itching and scratching symptoms had died, while other rabbits without any itching symptoms had survived, which indicated the inevitable relationship between scratching syndrome and death, again reflecting the high virulence of the PRV SD-2017 strain. H&E staining indicated that the brain was the main target organ, which is consistent with results in pigs, and the main pathological damage was meningitides, chromatolysis, and satellite phenomena (Figure 7A, PRV-challenged). Alveolar interstitial hyperplasia was observed in the lungs (Figure 7C, PRV-challenged), and inflammatory cell infiltration was observed in the liver (Figure 7B, PRV-challenged). The construction of a PRV SD-2017 strain-infected rabbit model contributes to improving the understanding of PR symptoms and to the development of fundamental PRV infection studies.

To obtain high purity gB and gD proteins and elicit a strong immune response in PRV-infected rabbits, we chose the Bac-to-Bac Baculovirus Expression System (BEVS), which can offer multifunctional proteins, laying the favorable foundations for the production of high-titer antibodies. In this study, gB and gD expression values were calculated for masses of 63 and 55 kDa, respectively (Figure 1), and the concentrations of gB and gD were 3 mg/mL and 300 μg/mL, respectively.

In our previous study, gB or gD individually was used to formulate subunit vaccines for immunization. Nonetheless, the single-protein subunit vaccine did not stimulate sufficient production of specific antibodies to protect rabbits from lethal PRV SD-2017 strain infection. Hence, we redesigned a protocol in which gB and gD in combination as subunit vaccines were used for immunization and achieved the expected outcomes in comparison to inactivated vaccines. Given the lower survival rate of rabbits treated with a dose of 50 μg of gD or gB in previous trials, we increased the immunization dose to 100 μg for rabbit immunization.

Subunit vaccines based on recombinant proteins are relatively stable compared with viral-vectored and whole virus vaccines and easy to produce by utilizing recombinant protein techniques, making them an attractive vaccine platform (Brisse et al., 2020). Moreover, subunit vaccines are not at risk of initiating disease owing to the absence of live-virion components, exhibiting excellent safety profiles (Vartak and Sucheck, 2016; Wang et al., 2016). Nevertheless, the advancement of subunit vaccines is seriously hindered by their shortcoming of low immunogenicity, thereby magnifying the role of adjuvants (Plotkin, 2014; Di Pasquale et al., 2015; Sarkar et al., 2019). Several *in vivo* studies have clearly indicated that GM-CSF could be developed into a novel class of immune enhancers (Zhao et al., 2018). Basta and Alatery (2007) found that GM-CSF increased tumor-specific antigen presentation to trigger excellent cross priming of T cells, playing a role as an adjuvant. Moreover, experiment-based findings originally demonstrated that GM-CSF is critical to produce T cells against various viral pathogens, such as EBV and HIV (Santodonato et al., 2003; Petrina et al., 2021). In our study, porcine-origin GM-CSF was expressed in the *E. coli* expression system. To verify the immunoenhancing activity of GM-CSF, ISA 201, which is a frequently used oil adjuvant for water-in-oil vaccine preparation, was selected for comparison. The results showed that the rabbits immunized with gB + gD + GM-CSF obtained better protection due to the higher titers of neutralizing antibodies (2^7.28^) than the rabbits immunized with gB + gD + ISA 201 (2^6.56^), inactivated PRV + GM-CSF (2^5.89^) and CIV (2^6.1^). The survival rate of the gB + gD + GM-CSF group (100%) was higher than that of the gB + gD + ISA 201 (80%), inactivated PRV + GM-CSF (60%) groups and CIV (60%) (Figures 4B,E), which is similar to the immune protection provided by gB + gD in mice reported by Zhang et al. (2020).

Because of the lack of a commercially available ELISA kit for quantification of anti-PRV gD antibodies, we used the recombinant gD protein that was expressed to coat the ELISA plate. After exploring the optimal conditions of the components, we constructed an indirect ELISA for anti-PRV gD antibodies to detect the anti-gD antibodies in rabbit serum (data unpublished). The results of gB and gD ELISAs showed that the gB- and gD-specific antibodies of all immunized groups were positive, and the antibody titer after the secondary immunization was higher than that after the first immunization (Figures 4C,D). Accordingly, the clinical symptoms were alleviated, and the lesions of organs were milder, which was consistent with the qPCR results (Figure 6) and the pathological analyses (Figure 7).

To further evaluate the immune efficacy of gB + gD + GM-CSF, IL-4, and IFN-γ were quantified to evaluate the cellular immune responses. IL-4 is vital for Th2 immunity, and IFN-γ is vital for Th1 immunity. Approximately 10.4 pg/mL of IL-4 was produced in response to gB + gD + GM-CSF immunization compared with gB + gD + ISA 201 immunization (9.2 pg/mL), indicating that GM-CSF promoted Th2-biased cellular immune responses, which suggested GM-CSF was mainly involved in promoting antibody-related immune responses. In addition, better Th1-biased cellular immune responses were produced by gB + gD + ISA 201 (137.6 pg/mL) compared with those induced by gB + gD + GM-CSF (127.2 pg/mL), which exhibited the better cellular and humoral immune efficacy of ISA 201 (Figure 5).

Additionally, the results of the gB + gD + GM-CSF group and inactivated PRV + GM-CSF group suggested that GM-CSF may be more suitable for subunit vaccines, which is related to the spatial structure of recombinant proteins, while the mechanism is unknown. Furthermore, the protective effect of gB + gD + GM-CSF was superior to that of gB + gD + ISA 201, and we inferred that GM-CSF promoted the maturation and differentiation of antigen presenting cells (APCs), strengthening the immune response. The better protection afforded by gB + gD + ISA 201 may be related to the prolonged duration of the antigen and slow release compared with those of inactivated PRV + GM-CSF. Additionally, the vaccine adjuvant GM-CSF is a water solution and easy to prepare, which are superior attributes compared to those of ISA 201.

PRV infects a wide range of animal species including swine as the natural host as well as ruminants, carnivores, rodents, and lagomorphs (Lian et al., 2020; Sehl and Teifke, 2020). Rabbits were found to be the most sensitive species compared to swine and mice (Hong et al., 2002). Also, typical PR symptoms such as itching are exhibited in rabbits and obvious changes can be observed between vaccinated rabbits, challenged rabbits, and normal rabbits. In this study, detailed tests were conducted about the efficacy of gB + gD + GM-CSF subunit vaccine, including antibodies, cytokines, viral load, and pathological examination, and efficacy of gB + gD + GM-CSF subunit vaccine was identified in rabbits. The choice of host is key to study the pathogenesis and pathophysiology for infectious diseases, however, rabbits are not the optimal host animal to PRV but swine, which is a limitation of this study, and further study should be undertaken to evaluate the efficacy of subunit vaccine in swine.

## Conclusion

In conclusion, gB + gD + GM-CSF subunit vaccines can provide comprehensive protection for rabbits compared with the inactivated PRV vaccines, and the adjuvant GM-CSF can remarkably enhance the immune effect compared with that for ISA 201, which implies that GM-CSF may be a preferred adjuvant candidate for subunit vaccines for the control and eradication of PR.

## Data availability statement

The original contributions presented in this study are included in the article/supplementary material, further inquiries can be directed to the corresponding author/s.

## Ethics statement

The animal study was reviewed and approved by Shandong SINDER Technology Co., Ltd. (SINDER, Shandong), and accordance with the protocols approved by the Animal Care and Ethics Committee of the SINDER, under number 20180024.

## Author contributions

ZC, KZ, and HLZ: data curation. HZ and YY: formal analysis. ZC, KZ, HZ, and DY: methodology. ZQ and HS: project administration. ZC and ZQ: supervision. ZC and KZ: writing – original draft. ZC, HS, and ZQ: writing – review and editing. All authors contributed to the article and approved the submitted version.
